# Therapeutic cancer vaccines for pediatric malignancies: advances, challenges, and emerging technologies

**DOI:** 10.1093/noajnl/vdab027

**Published:** 2021-02-11

**Authors:** Hannah E Olsen, Geoffrey M Lynn, Pablo A Valdes, Christian D Cerecedo Lopez, Andrew S Ishizuka, Omar Arnaout, W Linda Bi, Pier Paolo Peruzzi, E Antonio Chiocca, Gregory K Friedman, Joshua D Bernstock

**Affiliations:** 1 Department of Neurosurgery, Brigham and Women’s Hospital, Harvard Medical School, Boston, Massachusetts, USA; 2 Avidea Technologies, Inc., Baltimore, Maryland, USA; 3 Department of Neurosurgery, Boston Children’s Hospital, Harvard Medical School, Boston, Massachusetts, USA; 4 Division of Pediatric Hematology and Oncology, Department of Pediatrics, University of Alabama at Birmingham, Birmingham, Alabama, USA; 5 Department of Neurosurgery, University of Alabama at Birmingham, Birmingham, Alabama, USA

**Keywords:** checkpoint inhibitors, experimental therapeutics, immunotherapy, pediatric cancer, vaccination

## Abstract

Though outcomes for pediatric cancer patients have significantly improved over the past several decades, too many children still experience poor outcomes and survivors suffer lifelong, debilitating late effects after conventional chemotherapy, radiation, and surgical treatment. Consequently, there has been a renewed focus on developing novel targeted therapies to improve survival outcomes. Cancer vaccines are a promising type of immunotherapy that leverage the immune system to mediate targeted, tumor-specific killing through recognition of tumor antigens, thereby minimizing off-target toxicity. As such, cancer vaccines are orthogonal to conventional cancer treatments and can therefore be used alone or in combination with other therapeutic modalities to maximize efficacy. To date, cancer vaccination has remained largely understudied in the pediatric population. In this review, we discuss the different types of tumor antigens and vaccine technologies (dendritic cells, peptides, nucleic acids, and viral vectors) evaluated in clinical trials, with a focus on those used in children. We conclude with perspectives on how advances in combination therapies, tumor antigen (eg, neoantigen) selection, and vaccine platform optimization can be translated into clinical practice to improve outcomes for children with cancer.

Cancer continues to be a major cause of morbidity and mortality in children and is the second leading cause of death before adolescence.^1^ The prognosis of pediatric cancer has markedly improved in recent years, with the average overall survival (OS) of pediatric cancers rising from 58% in 1975 to 83% in 2014.^2^ Therapies for pediatric cancer, however, often cause significant toxicity that leads to lifelong disability.^3,4^ Additionally, many children will develop aggressive malignancies refractory to maximal medical and surgical management and ultimately experience dismal outcomes.^5^ Central nervous system (CNS) malignancies are among the most aggressive of pediatric cancers and are notoriously difficult to manage.^6,7^ Decades of research into tumor biology and clinical studies evaluating chemotherapy, radiation, and surgical resection-based approaches have had limited success in improving survival outcomes.^6,8^ The current standard of care for pediatric high-grade gliomas (HGGs), which represent approximately 10% of all pediatric CNS tumors, comprises surgical resection followed by concomitant radiotherapy and chemotherapy (ie, temozolomide) with a median OS of 10–18 months.^9–11^ Highly targeted agents with more favorable toxicity profiles and improved therapeutic efficacy are urgently needed to improve the quality of life and long-term outcomes of children with cancer.^12^

Immunotherapy harnesses the ability of the immune system to combat infection and neoplasia and has emerged as a promising treatment modality for many pediatric and adult malignancies.^12,13^ Cancer immunotherapy can be broadly defined as any therapy that leverages autologous or engineered immune cells to mediate tumor killing and, as such, encompasses a variety of treatments with diverse compositions and mechanisms of action.^14,15^ Examples of successful immunotherapies include antibodies that block immunosuppressive pathways, such as pembrolizumab, a PD-1 immune checkpoint inhibitor that has gained approval for treating a myriad of solid tumors; talimogene laherparepvec, an oncolytic virus approved for treating melanoma; and, more recently, tisagenlecleucel—a chimeric antigen receptor (CAR) T-cell therapy approved for treating pediatric acute lymphoblastic leukemia.^16–18^

Our understanding of tumor immunology has grown in tandem with technical improvements in vaccine development, leading to a renewed focus on vaccination as cancer therapeutic.^15,19–21^ Prophylactic vaccination against microorganisms (eg, smallpox, polio, and influenza) is a highly effective method of preventing life-threatening infections.^22^ Vaccination relies on exposing patients to the target microorganism or a structural fragment (ie, antigen) to generate an immune response that protects against future infection.^23^ Similarly, therapeutic cancer vaccines can induce immune cells, particularly cytotoxic CD8^+^ T cells, to become activated, expanded, and licensed to mediate tumor-specific killing through recognition of tumor antigens.^24^ As tumor cells demonstrate much greater homology with healthy tissue than infectious microorganisms, the crux of successful cancer vaccine construction lies in safeguarding healthy tissue while inducing targeted immunity against tumor antigens preferentially expressed by tumor cells.^20,21^

Tumor antigen selection is critical to the success of any cancer vaccine. The tumor antigen should be highly expressed and have a high affinity for binding major histocompatibility complex (MHC) molecules, which are key factors for ensuring that the antigen is adequately presented to enable immune cell recognition and killing.^25,26^ Additionally, the length and number of antigens must be considered to maximize the breadth of both CD8^+^ and CD4^+^ T-cell responses, which each have unique requirements for antigen recognition: CD8^+^ T cells recognize peptides of 8–11 amino acids in length presented in MHC-I, whereas CD4^+^ T cells recognize peptides of 12–15 amino acids in length bound to MHC-II.^27,28^ Several different classes of tumor antigens exist that can meet these criteria. Here we discuss the unique advantages and challenges associated with each class of tumor antigens.

Tumor-associated antigens (TAAs) are native proteins that are quantitatively overexpressed by tumor cells.^20,29^ The advantage of TAAs is that they tend to be upregulated (ie, highly expressed) by certain tumor types and are thus conserved targets for vaccination.^29^ Though the majority of vaccine studies have evaluated TAAs, this class of antigen faces a number of limitations. A key challenge is that TAA-reactive T cells can be removed by central tolerance, leaving only T cells with low affinity for tumor antigen recognition.^30^ Additionally, there is a possible risk of damage to healthy tissue; although off-target toxicity has not yet been reported in vaccine studies, CAR T cells targeting certain TAAs have resulted in severe, dose-limiting toxicities.^31,32^

In contrast to TAAs, tumor-specific antigens (TSAs)—also known as neoantigens, are non-autologous proteins (ie, mutated proteins) arising from tumoral genetic instability.^33^ TSAs provide the advantages that they are not expressed by healthy cells and are not subject to central tolerance. As compared with TAAs, TSAs generate more targeted and higher affinity T cells, thus providing potentially more effective treatment with a lower risk of autoimmunity.^34^ While several hotspot mutations have been identified leading to commonly occurring TSAs for certain tumor types^35,36^ as well as shared frameshift mutations occurring in microsatellite unstable tumors,^37^ most TSAs are highly variant between individuals.^38,39^ Thus, off-the-shelf approaches for targeting TSAs may not be practical for most patients and will instead require patient-specific identification and selection of TSAs for use in personalized cancer vaccines.^40,41^ Fortunately, with the advent and increasing availability of high-throughput molecular and genomic profiling, TSAs have become practicable targets for cancer vaccination.^42,43^

The feasibility and safety of TSA-directed vaccination have been demonstrated in adult melanoma trials in which a TSA-directed vaccine alone or in conjunction with checkpoint blockade therapy yielded a robust clinical response.^44^ Importantly, TSA-directed vaccination has also shown some efficacy against glioblastoma (GBM), despite this tumor’s relatively low mutation load and immune-privileged environment.^45,46^ Thus, these early trials provide compelling support for the use of TSA-directed vaccination in the pediatric cancer population, particularly for treating CNS malignancies.

In this review, we discuss the different classes of cancer vaccines and antigen targets while emphasizing their application in pediatric oncology. Vaccine classes are divided according to their composition and include cellular^47^ (eg, dendritic cells^48^), peptide,^49^ nucleic acid^50^ (DNA or RNA), and viral vector-based.^15,51^ Each has unique advantages and drawbacks as summarized in Table 1. As the majority of vaccination studies have been performed in adults, the utility and efficacy of this approach for pediatric malignancies, which can differ markedly from their adult counterparts in terms of molecular characteristics, histology, mutational burden, and neoantigen profile, has yet to be determined.^52–54^ We provide an overview of past and current challenges faced in vaccine development and conclude by highlighting emerging technologies that overcome historic challenges and therefore have greater promise for treating childhood malignancies.

**Table 1.** A Focused Comparison of the Different Classes of Cancer Vaccinations

Vaccination Class	Advantages	Disadvantages
Dendritic cell (DC)	• Clinical efficacy established (eg, Sipleucel-T) • Greater control over DC activation and phenotype through ex vivo manipulation	• High cost, labor-intensive manufacturing to process patient samples ex vivo
Peptide	• Synthetic and rapidly manufacturable using automated equipment • Most modular, enabling immune programming through adjuvant selection • Low toxicity, low risk for biocontamination	• Weakly immunogenic unless adequately formulated with immunostimulant (adjuvant) in nanoparticles • Limited number of antigens compared with nucleic acid and viral approaches
Nucleic acid (DNA/RNA)	• Rapid manufacturing using primarily automated equipment • RNA has inherent innate immune (adjuvant) activity, shown to lead to robust T-cell responses	• DNA weakly induces T-cell immunity • T-cell responses with RNA have been variable and depend on delivery platform and route of injection
Viral vectors	• Most potent vaccines for inducing T-cell immunity • Viruses with large genomes can accommodate many antigens and other encoded therapies	• Anti-vector immunity limits the number of injections that can be given to patients • Use of cell-based expression systems leads to higher costs, potential biocontaminants

## Vaccination Types

### Dendritic Cell Vaccines

Dendritic cells (DCs) are “professional” antigen-presenting cells (APCs) that are specialized for processing and presenting antigens for priming CD4^+^ and CD8^+^ T cells and producing cytokines that drive expansion and differentiation of T-cell responses.^55^ Thus, DCs act as a bridge between innate and adaptive immunity.^20,56,57^ For use as cellular vaccines, autologous DCs are isolated via apheresis, matured using immunostimulatory agents (eg, filgrastim), and loaded with an antigen before reinjection into the patient via the intradermal, intravenous, intranodal, or subcutaneous route.^58,59^ The advantage of isolating and manipulating DCs ex vivo is that antigen loading and activation can be performed under controlled conditions. Advances in gene-editing technologies including viral transduction, RNA interference, and CRISPR/Cas9 have greatly expanded scientists’ ability to engineer DCs to optimally perform a host of antitumoral functions.^60^ In addition, DC vaccines are often paired with adjuvants, such as imiquimod, interleukin-2, or KLH, which may be provided in the DC cultures or concomitantly delivered to increase the magnitude and duration of the antitumor response.^61^ Limitations of DC-based vaccines include the labor-intensive and costly cell isolation/enrichment and ex vivo stimulation process^62^, potentially weak responses due to insufficient cell numbers or inadequate cell activation or phenotype; and/or T-cell inactivation from an immunosuppressive tumor environment.^63^ Despite these challenges, it is notable that the first cancer vaccine to receive Food and Drug Administration approval is the DC vaccine sipuleucel-T (Provenge) for prostate cancer.^64^

Based on data from phase I/II trials, DC-based vaccination appears to be well tolerated with minimal toxicity in children with a diverse spectrum of malignancies and pretreatment conditions.^57,65–67^Table 2 provides a summary of DC-based vaccine trials in children. Of note, most of these trials used autologous whole tumor lysate as antigenic material. Despite the non-randomized format of early-phase safety studies, several pediatric trials of DC-based vaccines have reported improvement in clinical outcomes, albeit transient, which directly correlated with the degree of cytotoxic T lymphocyte (CTL) response as compared to historical controls. However, it should be noted that all patients eventually progressed on therapy. A phase II trial investigating an autologous tumor lysate-pulsed DC vaccine in children with metastatic or relapsed sarcomas demonstrated a 12% increase in OS in the DC-based vaccine group compared to children receiving standard of care chemotherapy. Five-year OS of patients in the Ewing sarcoma/rhabdomyosarcoma subgroup was an unprecedented 77%^68^ compared with 30–50% as per historical controls.^69–71^ Infusion of autologous DCs and CTLs has also been shown to eradicate the minimal residual disease in children with acute myeloid leukemia following chemotherapy.^72^ A study evaluating an autologous tumor lysate-pulsed DC vaccine in 45 children with relapsed malignant brain tumors demonstrated efficacy.^57^ Median OS for relapsed HGG, GBM, and anaplastic astrocytoma patients was 13.5, 12.2, and 18.4 months, respectively, with the authors noting that HGG and atypical teratoid–rhabdoid tumor appeared to respond more favorably as compared to medulloblastoma/primitive neuro-ectodermal tumor and ependymoma. A follow-up phase IIb trial is currently ongoing (EudraCT 2009-018228-14). Benitez-Ribas recently performed a phase I study of a DC vaccine pulsed with autologous tumor cell-lines lysate as the antigen administered intradermally with KLH as the adjuvant, which is used as a source of CD4^+^ T-cell (helper) epitopes to augment the response, in 9 patients with newly diagnosed diffuse midline glioma (DMG) (formerly termed “diffuse intrinsic pontine glioma” or “DIPG”), a highly aggressive and universally fatal subgroup of pediatric HGG.^73^ A specific antitumor response was observed in 8 patients as identified by immunologic studies in peripheral blood mononuclear cells. Cerebrospinal fluid (CSF) analyses showed anti-DMG specific T lymphocytes in 2 patients. Though the authors have not yet reported clinical outcomes data, the vaccine was well tolerated and no dose-limiting toxicities were observed.

**Table 2.** Trials of DC-Based Vaccines in Pediatric Patients

NCT	Study Phase	Tumor Type	Vaccine composition		Outcomes	Reference
			Antigen	Adjuvant		
*CNS*						
n/a	I	Recurrent brain tumors	Autologous RNA-pulsed DCs	None	PR (1/7), SD (2/7)	Caruso et al., 2004^65^
n/a	I	Relapsed malignant glioma	Autologous tumor peptide-pulsed DCs	None	RD patients (6): PR (1), SD (1)	Rutkowski et al., 2004^74^
					CR patients (6): CCR 3 years (2)	
n/a	Not specified	Recurrent malignant brain tumors	Autologous whole tumor lysate-pulsed DCs	Imiquimod	OS:	Ardon et al., 2010^57^
					HGG: 13.5 m GBM: 12.2 m AA: 18.4	
NCT00107185	I	Newly diagnosed or recurrent HGG	Autologous whole tumor lysate-pulsed DCs	None	PR (1/3), SD (2/3)	Lasky et al., 2013^75^
NCT02840123	I	New diagnosed DIPG	Autologous DCs pulsed with allogeneic tumor lysate	None	No data	Benitez-Ribas et al., 2018^73^
*Solid tumor*						
NCT00405327	II	Relapsed solid tumors	Autologous tumor peptide-pulsed DCs	KLH	PR (1/15), SD (5/15)	Geiger et al., 2001^66^
		Recurrent alveolar rhabdomyosarcoma and Ewing sarcoma	Autologous tumor peptide (breakpoint region of fusion protein)-pulsed DCs	IL-2	PD (15/15)	Dagher et al., 2002^76^
n/a	Not specified	Advanced solid extra-cranial tumors	Autologous tumor lysate-pulsed DCs	KLH	SC-treated patients (14):	Dohnal et al., 2007^77^
					- CR patients (5): CCR (4), SD (1)	
					- PR patient (1): PD (1)	
					- PD patients (8): MR (1), SD (1)	
					IN-treated patients (8):	
					- CR patients (4): CCR (3), PD (1)	
					- PD patients (4): PD (4	
NCT00001566	II	Metastatic or recurrent Ewing sarcoma and alveolar rhabdomyosarcoma	Autologous tumor peptide (translocation breakpoint)-pulsed DCs	IL-2	CR (17/30), PR (11/30), PD (2/30)	Mackall et al., 2008^78^
n/a	Not specified	Refractory Ewing sarcoma, synovial sarcoma, neuroblastoma	Autologous tumor lysate-pulsed DCs	KLH	CR (1/5), SD -> PD (2/5), PD (1/5)	Suminoe et al., 2009^79^
n/a	I	Relapsed osteosarcoma	Autologous tumor lysate-pulsed DCs	KLH, IL-2	No evidence of tumor regression	Himoudi et al., 2012^80^
NCT01241162	I	Relapsed/refractory solid tumors neuroblastoma and sarcoma	Autologous tumor peptide (MAGE-A1, MAGE-A3, and NY-ESO-1 derived)-pulsed DCs	Imiquimod	CR (1/10), SD (1/10)	Krishnadas et al., 2015^81^
NCT00923351	I/II	Metastatic and relapsed high-risk sarcomas	Autologous tumor lysate-pulsed DCs	KLH, IL-7	OS:	Merchant et al., 2016^68^
					-Overall 51%	
					-ES/RMS: 63%	
*Hematological*						
n/a	Not specified	AML	Autologous DCs and cytokine-induced killer cells	IL-2	CR (20/22), PD (2/22)	Bai et al., 2015^72^
n/a	Case report	Relapsed ALL	Allogeneic WT-1-pulsed DCs	OK-432	Relapse 14 months following treatment	Saito et al., 2015^67^
NCT00923910	I/II	Post-HSCT relapsed ALL, AML, HL	Allogeneic WT-1-pulsed DCs	KLH	PD (5/5)	Shah et al., 2016^82^

AA, anaplastic astrocytoma; ALL, acute lymphoblastic leukemia; AML, acute myeloid leukemia; CNS, central nervous system; CR, complete response; ES, Ewing sarcoma; GBM, glioblastoma multiforme; HGG, high-grade glioma; HL, Hodgkin’s lymphoma; HSCT, hematopoietic stem cell transplantation; IN, intranodal; KLH, Keyhole limpet hemocyanin; MR, mixed response; PD, progressive disease; PR, partial response; OS, overall survival; RD, residual disease; RMS, rhabdomyosarcoma; SC, subcutaneous; SD, stable disease; WT-1, Wilms’ tumor 1.

In summary, DC-based vaccination appears to be well tolerated and effective for inducing T-cell immunity in pediatric cancers, though additional studies will be needed to fully understand the potential of these therapies for the pediatric population.

### Peptide Vaccines

An alternative to loading DCs with antigenic material ex vivo is to vaccinate patients with peptide antigens that can be processed and presented by endogenous APCs, particularly DCs, that prime T-cell immunity in lymph nodes draining the sites of vaccination.^49^ Peptide vaccines generally include one or more synthetic peptides comprising tumor antigens combined with immunostimulants (“adjuvants”) that are used to enhance peptide antigen immunogenicity.^83,84^ The length of peptide antigens is selected to maximize the breadth of the T-cell response and depends, in part, on the antigenic target. For TSAs, peptide antigens of 25 amino acids in length are typically used wherein the middle (15th amino acid) is the mutant (ie, tumor-specific) residue.^85,86^ This length ensures that all 8–11 amino acid CD8^+^ T-cell epitopes and most 12–15 amino acid CD4^+^ T-cell epitopes including the mutant amino acid are represented in each sequence. A similar rationale is applied to TAAs, whereby protein antigens greater than 100 amino acids in length are produced as a pool of peptide fragments overlapping by 9–14 amino acids to ensure that most CD4^+^ and CD8^+^ T-cell epitopes are represented.^87,88^ Finally, to maximize the breadth of T-cell responses, peptide vaccines typically composed of up to 20 unique peptide antigens, which is largely dictated by manufacturing and cost constraints.

Peptide vaccines are advantageous in that they can be produced rapidly, at relatively low cost, entirely by synthetic processes using automated equipment.^89,90^ Additionally, peptide vaccines are among the most modular as they, unlike, other vaccine platforms (eg, viruses), have little to no inherent immunostimulatory properties and therefore enable the quality and magnitude of the immune response to be programmed based on the adjuvant(s) used.^91–93^ A potential disadvantage of peptide-based vaccines is that they permit a lower antigen payload (~20 peptide antigens each of 25–35 amino acids in length) as compared with recombinant vaccine approaches (eg, viruses and nucleic acids) that can encode multiple protein antigens that are each several hundred amino acids in length.^94^ However, more antigens are not necessarily better. The presence of multiple antigens can lead to competition that may diminish the response against any one specific antigen.^20,34^ Additionally, T-cell responses directed against a single antigen can mediate durable tumor regression.^95,96^ Thus, it is likely that the antigen payload of peptide-based therapeutic cancer vaccines, which typically comprise about 10–20 peptide antigens, is sufficient given appropriate antigen selection.^43,97^

Table 3 provides a summary of peptide-based vaccine trials in children. In the pediatric setting, several early-phase studies have investigated Wilms’ Tumor gene, WT-1, targeted peptide-based vaccines in children with solid and hematologic malignancies. In a study of 5 patients with various malignancies, Hashii et al.^98^ found that intradermal vaccination with a single WT-1-derived short (9 amino acid) peptide antigen formulated in a water-in-oil emulsion-based adjuvant, Montanide ISA51, induced complete remission in one patient and a period of stable disease in another patient. However, clinical benefit was limited with 4 patients experiencing disease progression and/or death during the trial. In a larger study of 26 children and young adults less than 20 years old with relapsed solid and hematologic malignancies, no patients demonstrated a clinical response.^99^ Limited efficacy with this approach may be due to the use of only a single short (minimal epitope) peptide antigen resulting in limited T-cell breadth, and/or use of an emulsion formulation that lacks immunostimulants (eg, Toll-like receptor agonists [TLRa]) needed for strong T-cell induction^100^ and may instead promote T-cell exhaustion.^101^

**Table 3.** Trials of Peptide-Based Vaccines in Pediatric Patients

NCT	Study Phase	Tumor Type	Vaccine Composition		Outcomes	Reference
			Antigen	Adjuvant		
*CNS*						
NCT01130077	I	High-risk gliomas	IL-13 receptor alpha 2, EphA2, survivin	Montanide ISA51, poly-ICLC	CR (2/24), PR (2/24), MR (1/24) SD (19/24)	Pollack et al., 2014^102^
NCT01130077	I	Recurrent low-grade gliomas	IL-13 receptor alpha 2, EphA2, survivin	Montanide ISA51, poly-ICLC	PR (4/24), MR (1/24) SD (7/24)	Pollack et al., 2016^103^
NCT02960230	I	DIPG, nonpontine DMG	H3.3K27M	Montanide ISA51, poly-ICLC	OS at 12 months DIPG 44%, nonpontine DMG 39%	Mueller et al., 2020^104^
*Solid tumor*						
n/a	II	Rhabdomyosarcoma, osteosarcoma, liposarcoma, synovial sarcoma	WT-1	Montanide ISA51	CR (1/4), SD (1/4), PD (2/4)	Hashii et al., 2010^98^
n/a	I/II	Relapsed/refractory solid tumors	WT-1	Montanide ISA51	CR (5/9), MR (1/9), SD (1/9), PD (2/9)	Sawada et al., 2016^99^
n/a	Not specified	Solid tumors	WT-1	OK-432	No data	Hirabayashi et al., 2018^105^
n/a	I	Neuroblastoma	NY-ESO-1	Montanide ISA51	No data	Camisaschi et al., 2018^106^
n/a	I	Refractory solid tumors	KOC1, FOXM1, KIF20A	Incomplete Freund’s adjuvant	SD (4/12), MR (2/12), PD (6/12)	Akazawa et al., 2019^107^
*Hematological*						
n/a	II	ALL	WT-1	Montanide ISA51	PD (1/1)	Hashii et al., 2010^98^
n/a	II	ALL, AML	WT-1	Montanide ISA51	CR (2/3), PD (1/3)	Hashii et al., 2012^108^
n/a	I/II	ALL, AML, lymphoma	WT-1	Montanide ISA51	CR (4/4)	Sawada et al., 2016^99^

ALL, acute lymphoblastic leukemia; AML, acute myeloid leukemia; CNS, central nervous system; CR, complete response; DMG, diffuse midline gliomas; MR, mixed response; PD, progressive disease; poly-ICLC, polyinosinic–polycytidylic acid complexed with poly(lysine) and carboxymethylcellulose; PR, partial response; OS, overall survival; SD, stable disease; WT, Wilms’ tumor 1.

Additional peptide vaccines targeting conserved tumor antigens have been tested in pediatric clinical studies. Kushner et al.^109^ performed a phase I trial in which 15 children with high-risk neuroblastoma were administered a vaccine containing the immunological adjuvant OPT-821 and the neuroblastoma-associated antigens GD2 and GD3. Patients were also given β-glucan, a biologic response modifier that enhances the antitumor response.^110,111^ No patients had dose-limiting toxicities and 12 patients demonstrated an antibody response against GD2 and/or GD3. Carcinoembryonic antigen glypican-3 (GPC3), a hepatic heparan sulfate proteoglycan expressed in many pediatric tumors such as hepatoblastoma, yolk-sac tumors, and Wilms’ tumors, has also been piloted as a target for vaccination.^112,113^ A phase I study of 18 pediatric patients with GPC3-expressing solid tumors found that vaccination with a single MHC-I matched GPC3-peptide formulated in a water-in-oil emulsion improved or maintained clinical status (CR + PR + SD) in 67% of patients,^114^ despite using a suboptimal emulsion formulation, as discussed above.

Early peptide vaccine studies in pediatric CNS malignancies have been encouraging. Pollack et al.^102^ investigated the safety and performance of a peptide vaccine targeting known glioma-associated antigens (IL-13Rα2, EphA2, and survivin) administered subcutaneously with polyinosinic–polycytidylic acid (poly[I:C]), a TLR-9 agonist, stabilized by poly(lysine) and carboxymethylcellulose (poly-ICLC) adjuvant in 26 children with newly diagnosed brainstem or non-brainstem gliomas. Five children had pseudoprogression, a transient increase in edema, and contrast enhancement secondary to a treatment-induced immune response that was followed by stabilization and/or regression.^115^ Patients with pseudoprogression were successfully treated with dexamethasone and had a higher median survival (19.5 months vs 10.9 months). A phase I study of cytomegalovirus (CMV)-specific peptide vaccine in patients with recurrent medulloblastoma and malignant glioma is ongoing (NCT03299309) and is of interest given the noted expression of CMV proteins in such tumors.^116^

Peptide vaccines are also being studied in DMG. Ochs et al.^117^ showed that vaccination with peptides derived from H3.3K27M (a unifying oncogenic mutation resulting in global methylation perturbation), and formulated in a water-in-oil emulsion administered subcutaneously, produced an effective, mutation-specific CD4^+^ and CD8^+^ T-cell-mediated immune response with antigen presentation on both MHC classes I and II in a humanized mouse model. Although they observed tumor regression in murine models, the experimental design consisted of DMG tumors in the flank rather than intracranially. This vaccine epitope is currently being tested in phase 1 clinical trial in combination with checkpoint inhibitors in children (NCT02960230).

While it is too early to quantify the potential of peptide-based vaccines for pediatric cancers, more advanced trials have been conducted in the adult population. Indeed, several phase III adult solid tumor trials have been conducted and have failed to show any clinical benefit.^118–120^ While these results have dampened the initial enthusiasm for peptide-based vaccine approaches, recent mechanistic data suggest that suboptimal formulations may account, in part, for the observed weak efficacy and that formulating peptide antigens in nanoparticles that target endogenous DCs that promote T-cell immunity may be needed to improve efficacy.^121^ Additionally, similarly to DC vaccines, peptide vaccines appear to perform optimally in patients with lower disease burden and when administered concomitantly with other treatment modalities like checkpoint inhibitors to allow for synergistic antitumor effects.^122^

In summary, peptide-based cancer vaccines can safely induce anticancer T-cell immunity in pediatric and adult populations; however, further studies are needed to understand how to optimize the composition (eg, delivery vehicle and adjuvant) and combination approaches with complementary therapeutic modalities to maximize T-cell responses and efficacy in children.

### Nucleic Acid Vaccines

Nucleic acid vaccines utilize plasmid DNA or mRNA to express tumor antigens through transient transfection of muscle tissue or APCs (eg, DCs) following administration. Multiple genes can be incorporated into a single vector, and the nucleic acid can be modified to modulate expression and innate stimulation to augment the immune response.^65,123,124^ Similar to peptide-based vaccines, nucleic acid vaccines enable lower costs and more rapid manufacturing as compared with cellular or viral vector-based vaccines, but, unlike peptides, still require the use of costly recombinant enzymes for production.^125^ While early clinical studies in adult patients showed that DNA vaccines were sufficient in invoking a cellular and humoral immune response, there was little evidence of clinical benefit.^126^ Indeed, recent preclinical data suggest that the platform and site of nucleic acid administration are critical to the capacity of such approaches to induce T-cell immunity.^127^ Thus, numerous delivery approaches have been developed to improve antigen expression in APCs as a means to improve efficacy through the use of direct intra-lymph node injection, gene gun, electroporation, ultrasound, laser, liposome, microparticles, and/or nanoparticles.^128–131^ Among the nucleic acid vaccine approaches, RNA lipoplexes appear to be one of the most promising and have shown the greatest capacity for inducing anticancer T-cell immunity.^127^ Though additional optimization will be needed to balance gene expression with innate stimulation that may lead to systemic toxicity and blunting of antigen expression, early data suggest that RNA-based vaccination approaches may eventually have a great therapeutic impact in pediatric cancers.

### Viral Vector Vaccines

Recombinant viral vectors, typically derived from the *Poxviridae*, *Adenoviridae*, and *Rhabdoviridae* families, are engineered to express tumor antigen transgenes and are among the most potent vaccine technologies for inducing T-cell immunity.^132–134^ Viral vectors provide the advantages that they can encode a large number of antigens that can be expressed at high levels and rendered immunogenic through intrinsic innate immunostimulatory capacity of the virus. Viral vectors can be further engineered to maximize gene expression, target specific cell populations, and/or encode multiple additional therapeutic modalities.^135^ Though viral vectors are easier to produce, purify, and store relative to more costly and labor-intensive cellular vaccines, like DC vaccines,^136^ their dependence on recombinant technologies can result in higher costs than synthetic peptide-based vaccine approaches, and antibodies generated against the vector (antivector immunity) can limit their use to a single administration.^137^

PROSTVAC, a well-studied poxviral-based vaccine targeting prostate-specific antigen that also contains transgenes for T-cell co-stimulatory molecule expression, showed promise in an early-phase II double-blind randomized trial in metastatic castration-resistant prostate cancer; however, results from the subsequent phase III trial failed to show any survival improvement over placebo.^138,139^ PROSTVAC in combination with the anti-CTLA-4 checkpoint inhibitor, ipilimumab, for metastatic castration-resistant prostate cancer has been proven safe in a phase I trial^140^ and is currently being assessed in a randomized phase II trial (NCT02933255).

The vaccine TG4010, a modified vaccinia Ankara vector expressing MUC1 and IL-2, has been evaluated in combination with first-line chemotherapy for the treatment of patients with advanced-stage non-small-cell lung cancer and was found to improve progression-free survival at 6 months.^141^ Though there are no current ongoing clinical trials, there are promising preclinical data to suggest that viral-based vaccines may be effective in brain tumors. Abdelaziz et al.^142^ designed human cytomegalovirus (HCMV)-based vaccine expressing E6-derived peptide fused to HCMV proteins. Patient-derived GBM cells infected with these vectors efficiently stimulated E6-specific T cells. Additionally, a phase I dose escalation evaluating the safety of aglatimagene besadenovec (AdV-tk), an adenoviral vector expressing herpes virus thymidine kinase, followed by anti-herpetic prodrug in pediatric malignant glioma or recurrent ependymoma found the approach to be safe in combination with radiation therapy and temozolomide^143^; a subsequent phase II study is planned.

## Combination Immunotherapy

Therapeutic cancer vaccines principally mediate tumor clearance through the induction of cytotoxic T cells. However, cancers can evade T-cell recognition and killing by promoting an immunosuppressive environment, including through the expression of immune checkpoint molecules (eg, PD-L1) that directly inhibit T-cell killing. To overcome the immunosuppressive environment of tumors, vaccines that induce T-cell immunity should be used in combination with complementary therapies that reverse immune suppression. Indeed, many groups are starting to explore the potential of a combinatorial approach using vaccination to enhance the efficacy of other immunotherapy-based treatment modalities including checkpoint inhibitors, antiangiogenic agents, oncolytic viruses, and radiation.^19,144^

One of the most promising combination immunotherapies is the use of cancer vaccines with checkpoint inhibitors (eg, anti-PD-1/PD-L1 and anti-CTLA-4). Myriad preclinical studies have shown that cancer vaccines used in combination with checkpoint inhibitors lead to significantly improved efficacy as compared with either treatment used alone,^145^ and now multiple clinical trials are ongoing evaluating this combination in patients.^19^ Among the most promising studies thus far, a phase I trial found that a GM-CSF cell-based vaccine (“GVAX”) in combination with ipilimumab, an anti-CTLA-4 antibody, enhanced the preexisting endogenous tumor-specific T-cell response compared to treatment with ipilimumab alone. Posttreatment expansion of the mesothelin-specific T-cell repertoire was associated with a significant improvement in OS in the combination arm, suggesting that the frequency of preexisting mesothelin-specific T cells are low and require a vaccine to induce larger pools of precursor T-cells.^146^

The use of viruses to modulate the tumor microenvironment and provide a more permissive environment for T-cell killing is another promising strategy that is gaining increasing attention. Accordingly, Koske et al.^147^ demonstrated that combination treatment of Vesicular Stomatitis Virus-glycoprotein (VSV-GP), a chimeric VSV pseudotyped with GP of the lymphocytic choriomeningitis virus, followed by an ovalbumin peptide-loaded DC vaccine, significantly enhanced survival over either agent alone in a murine melanoma model. The authors found that this strategy alleviated local immune suppression in the tumor microenvironment by reducing regulatory T cells, activating tumor-infiltrating lymphocytes, and increasing inflammatory cytokines.

Though most combinatorial approaches have been tested in adults, there are a number of ongoing trials in the pediatric setting. A phase I trial using GVAX in combination with nivolumab (anti-PD-1) and ipilimumab (anti-CTLA-4) for refractory neuroblastoma is ongoing (NCT04239040). Krishnadas et al. reported complete remission in a patient with relapsed stage 4 neuroblastoma after treatment with decitabine to upregulate cancer testis antigen expression, followed by DC vaccine targeting the cancer testis antigens MAGE-A1, MAGE-A3, and NY-ESO. Additional phase I studies are underway to evaluate concomitant vaccination and radiation for pediatric HGG (NCT03615404, NCT02722512, and NCT00634231).

## Challenges and Emerging Technologies

While a variety of cancer vaccines have been evaluated in adult and pediatric patients, most have provided only modest efficacy. The shortcomings of prior vaccines may be in part due to weak immunogenicity of the vaccine leading to insufficient magnitude of CD8^+^ T-cell responses; inadequate, or loss, of expression of the tumor antigen, thereby enabling tumor cells to evade recognition; and/or, inability of vaccine-induced T cells to overcome the suppressive tumor microenvironment.^15,20,148^ Recent advances in tumor antigen identification and selection algorithms are enabling more reliable antigen selection, and checkpoint inhibitors have proven effective for unleashing T cells to mediate tumor-specific killing. A key challenge remains the availability of vaccine technologies for reliably inducing high-magnitude CD8^+^ T-cell responses that correlate with immunotherapy efficacy.^15,20,148^ Indeed, patients treated with a peptide-based vaccine comprising peptide neoantigens admixed with the immunostimulant polyICLC (Hiltonol) experienced limited benefit from therapy despite promising preclinical data.^85^ Weak efficacy may be, in part, attributed to inadequate formulation of the peptide neoantigens (see below), though limitations in neoantigen prediction and selection cannot be ruled out as their validation is not possible in the absence of clear vaccine-mediated efficacy.

Toward improving peptide-based vaccine formulations for inducing CD8^+^ T-cell immunity, Lynn et al.^121^ developed a vaccine platform based on peptide antigen–TLRa conjugates that are programmed to self-assemble into nanoparticles of an optimal size (~20 nm) for targeting lymph node resident DCs that promote T-cell immunity. Programmed self-assembly was developed to ensure formulation consistency for all possible antigens that can be generated from the human genome, thus enabling a universal approach for formulating tumor antigens. Importantly, improved formulation of tumor antigens, including neoantigens, with TLRa in self-assembling nanoparticles promoted improved uptake by and activation of APCs (eg, DCs) that was associated with enhanced immunogenicity and improved tumor clearance in 3 murine tumor models.^121^ They also found that peptide physical form is a key determinant of CD8^+^ T-cell immunogenicity.

Specifically, they showed that hydrophilic, water-soluble peptide antigens are often non-immunogenic even when combined with potent immunostimulants, such as polyICLC, but that rendering such peptide antigens particulate significantly improves immunogenicity.^121^ These data suggest that codelivery of peptide antigens with specific immunostimulants in nanoparticles will be key to the success of peptide-based cancer vaccines and that self-assembling nanoparticles (eg, SNP-7/8a) offer an effective platform for achieving consistent nanoparticle formulations needed for reliable induction of CD8^+^ T cells.

Another promising peptide-based vaccine approach for ensuring codelivery of tumor antigens and immunostimulants in nanoparticles for inducing T-cell immunity is the use of synthetic high-density lipoprotein nanodiscs, which can be coupled with immunostimulatory CpG oligonucleotides and tumor antigen peptides. Kuai et al.^149^ recently reported that the use of this nanocarrier technology in a murine colon adenocarcinoma model generated a robust neoantigen-specific CD8^+^ T-cell response resulting in complete inhibition of tumor growth; additionally, concomitant vaccination with dual PD-1 and CTLA-4 blockade led to complete tumor regression in approximately 90% of mice.

Though many next-generation vaccine technologies have been focused on solid tumors, innovation in biomaterials science has also made considerable progress in the setting of hematologic malignancies. Shah et al.^150^ developed a macroporous cryogel composed of cross-linked polyethylene glycol and alginate scaffolding with entrapped TLR-9a cytosine–guanosine oligodeoxynucleotide and GM-CSF immunostimulants. Prophylactic administration of this vaccine with either WT-1 antigen or tumor cell lysate in a mouse model of AML elicited a potent CTL response and prevented engraftment of malignant cells in the bone marrow. Combinatorial administration of the vaccine with standard chemotherapeutic agents eradicated established AML and generated transferable protective T-cell immunity.

In addition to the type of vaccine used, the route of administration and vaccine schedule will likely require optimization in the clinical setting to achieve maximal benefit for patients. Accordingly, while most vaccines are administered by the intramuscular or subcutaneous routes, mounting evidence suggests that vaccination by the intravenous route may be favorable for promoting T-cell-mediated immunity independent of the vaccination platform used.^127,151^

A final consideration is how to integrate cancer vaccines into a complex treatment regimen comprising chemotherapy, radiotherapy, and immunotherapy, which may not always have synergistic effects. Indeed, many cancer patients require corticosteroid therapy at different points throughout treatment for various reasons (eg, tumor/treatment-related edema, pain relief, appetite stimulation).^152^ However, there is growing evidence to suggest that the immunosuppressive nature of these drugs, due in large part to their effects on T-cell apoptosis, may reduce the efficacy of therapeutic approaches that rely on stimulating a robust anticancer immune response.^153,154^ Wong et al.^155^ found that dexamethasone treatment in adult patients with recurrent GBM profoundly decreased the efficacy of radiation and chemotherapy, leading to lower OS. Pitter et al.^156^ found similar results in a retrospective analysis of GBM patient cohorts, which showed dexamethasone-induced antiproliferative effects conferred protection from radiotherapy- and chemotherapy-induced genotoxic stress. In a phase I study of H3.3K27M peptide vaccination in pediatric patients with DMG and DIPG, Mueller et al.^104^ found a negative association between dexamethasone administration and the longitudinal expansion of vaccine-reactive CD8^+^ T cells. Though, while immunosuppressive chemotherapy agents can have deleterious effects on cancer vaccine efficacy, certain chemotherapy regimens, particularly those that promote immunogenic cell death or selective depletion of suppressor cells (eg, regulatory T cells or myeloid-derived suppressor cells), have been shown to enhance vaccine efficacy.^157,158^ Therefore, further research will be needed to fully delineate the impact and optimal regimen of chemotherapies, including corticosteroids, on cancer vaccination efficacy.

## Conclusions

The ability of checkpoint inhibitors and adoptive cell therapies to mediate durable regression of certain advanced cancers provides clinical proof-of-concept that tumor antigen-specific T cells can mediate tumor clearance and improve patient outcomes. These observations have fueled a resurgence in efforts to advance therapeutic cancer vaccines for priming and/or expanding tumor antigen-specific T-cell responses in patients for use alone or in combination with other therapies. Despite their immense promise, however, therapeutic cancer vaccines have only shown modest benefit in a small cohort of primarily adult patients in early-stage trials. While many challenges remain, the emergence of improved vaccine technologies for inducing T-cell immunity, as well as refined tools for tumor antigen selection, provide optimism that next-generation therapeutic cancer vaccines may effectively overcome historic limitations. Additionally, combination immunotherapies, including vaccines combined with checkpoint inhibitors, oncolytic viruses, certain chemotherapeutics,^159^ and/or radiation, are emerging as effective approaches for reversing immune suppression within the tumor and augmenting vaccine efficacy.

Finally, it should be noted that vaccines and other immunotherapies that are safe but fail to demonstrate efficacy in adults with advanced cancers should not be ruled out for evaluation in pediatric populations. Adults are less responsive to vaccines due to thymic atrophy, whereas younger patients are more responsive to immunotherapies and are therefore more likely to mount an effective T-cell response to therapeutic cancer vaccines. Despite this general recognition, most novel immunotherapies are evaluated in adult populations, and treatments that fail in adults are often not advanced to testing in children. However, the small number of early-phase studies that have been conducted with pediatric patients has shown great promise in terms of safety, feasibility, and ability to generate an immunologic response. Though objective clinical response rates are low, advances in antigen design, adjuvant therapy, and combinatorial approaches may drastically change the landscape of immunotherapy in pediatric cancer.

In summary, given the tremendous potential of therapeutic cancer vaccines and their higher likelihood of success in pediatric populations, cancer vaccines and combination therapies should be rigorously investigated as potentially life-saving treatments for children with advanced malignancies refractory to conventional approaches.
